# New criteria for diagnosis of benign joint hypermobility in children

**DOI:** 10.1186/1546-0096-12-S1-P98

**Published:** 2014-09-17

**Authors:** Reza Shiari, Vadood Javadi Parvaneh

**Affiliations:** 1 Pediatric Rheumatology, Mofid Children's Hospital, Tehran, Iran, Islamic Republic Of

## Introduction

Benign joint hypermobility (BJH) is a clinical condition characterized by an increased ability of joints during passive and dynamic movements.

## Objectives

To our knowledge, current criteria's for BJH are on the basis of all age groups. These criteria are not specified for pediatrics yet. Therefore, we decided to determine new diagnostic criteria for benign hypermobility in children and compared it with the most popular, Beighton Criteria.

## Methods

Clinical study which enrolled 108 cases, from 3 to 16 years of age who was diagnosed as BJH previously by Beighton Criteria. The children separately examined by two qualified pediatric rheumatologists and the Beighton score for hypermobility was calculated again; eight children were excluded from the study during re-exams and for remainders (100 cases) examinations were performed based on the new criteria and the questionnaires filled out.

## Results

: Data analysis revealed a significant correlation between Beighton and new criteria in a way that Pearson correlation, Interclass correlation and P values were 0.685, 0.582 and 0.001 respectively. The mean, median, minimum, maximum and standard deviation of new scores was 6.55, 7, 4, 8 and 1.11 separately.

## Conclusion

The new criteria are able to detect cases of benign joint hypermobility in pediatric age group. Although for assess the criteria power in order to differentiate non-hypermobile from hypermobile items, we need to carry out another study in normal children groups (according to Beighton criteria) that is currently in progress.

## Disclosure of interest

None declared.

